# Determining Line of Therapy from Real‐World Data in Non‐Small Cell Lung Cancer

**DOI:** 10.1002/pds.70049

**Published:** 2024-11-25

**Authors:** Connor B. Grady, Wei‐Ting Hwang, Joshua E. Reuss, Wade Iams, Amanda Cass, Geoffrey Liu, Devalben Patel, Stephen V. Liu, Gabriela Liliana Bravo Montenegro, Tejas Patil, Jorge J. Nieva, Amanda Herrmann, Kristen A. Marrone, Vincent K. Lam, William Schwartzman, Jonathan Dowell, Liza C. Villaruz, Kelsey Leigh Miller, Jared Weiss, Fangdi Sun, Vamsidhar Velcheti, D. Ross Camidge, Charu Aggarwal, Lova Sun, Melina E. Marmarelis

**Affiliations:** ^1^ Department of Biostatistics, Epidemiology and Informatics Perelman School of Medicine Philadelphia Pennsylvania USA; ^2^ Department of Medicine Georgetown University Washington DC USA; ^3^ Division of Hematology/Oncology, Department of Medicine Vanderbilt University Medical Center Nashville Tennessee USA; ^4^ Department of Medical Oncology and Hematology Princess Margaret Cancer Centre Toronto Ontario Canada; ^5^ Division of Medical Oncology, Department of Medicine University of Colorado Cancer Center Aurora Colorado USA; ^6^ Department of Medicine University of Southern California/Norris Comprehensive Cancer Center Los Angeles California USA; ^7^ Department of Oncology Johns Hopkins University School of Medicine Baltimore Maryland USA; ^8^ Department of Internal Medicine Harold C Simmons Comprehensive Cancer Center, UT Southwestern Dallas Texas USA; ^9^ Department of Medicine UPMC Hillman Cancer Center Pittsburgh Pennsylvania USA; ^10^ Department of Medicine Lineberger Comprehensive Cancer Center Chapel Hill North Carolina USA; ^11^ Department of Medicine UCSF School of Medicine, University of California San Francisco San Francisco California USA; ^12^ Department of Medicine NYU Grossman School of Medicine New York New York USA; ^13^ Department of Medicine, Division of Hematology and Oncology Perelman School of Medicine, University of Pennsylvania Philadelphia Pennsylvania USA

**Keywords:** ALK, EGFR, non‐small cell lung cancer, real‐world data, ROS1

## Abstract

**Introduction:**

Determining lines of therapy (LOT) using real‐world data is crucial to inform clinical decisions and support clinical research. Existing rules for determining LOT in patients with metastatic non‐small cell lung cancer (mNSCLC) do not incorporate the growing number of targeted therapies used in treatment today. Therefore, we propose rules for determining LOT from real‐world data of patients with mNSCLC treated with targeted therapies.

**Methods:**

LOT rules were developed through expert consensus using a real‐world cohort of 550 patients with *ALK*+ or *ROS1*+ mNSCLC in the multi‐institutional, electronic medical record‐based Academic Thoracic Oncology Medical Investigators Consortium's (ATOMIC) Driver Mutation Registry. Rules were subsequently modified based on a review of appropriate LOT determination. These resulting rules were then applied to an independent cohort of patients with *EGFR*+ mNSCLC to illustrate their use.

**Results:**

Six rules for determining LOTs were developed. Among 1133 patients with *EGFR* mutations and mNSCLC, a total of 3168 regimens were recorded with a median of 2 regimens per patient (IQR, 1–4; range, 1–13). After applying our rules, there were 2834 total LOTs with a median of 2 LOTs per patient (IQR, 1–3; range, 1–11). Rules 1–3 kept 11% of regimen changes from advancing the LOT. When compared to previously published rules, LOT assignments differed 5.7% of the time, mostly in LOTs with targeted therapy.

**Conclusion:**

These rules provide an updated framework to evaluate current treatment patterns, accounting for the increased use of targeted therapies in patients with mNSCLC, and promote standardization of methods for determining LOT from real‐world data.

**Summary**

- Use of targeted therapy to treat patients with mNSCLC is growing.
- Determining lines of therapy from real‐world data is crucial for clinical research.
- Our rules aim to advance the line of therapy to reflect changes in clinical status.
- Using these rules can lead to better method harmonization in mNSCLC research.

## 1 Introduction

When using real‐world data to answer clinical questions in oncology, accurate determination of lines of therapy (LOT) using treatment records is crucial to conducting meaningful analyses. In the absence of detailed information on progression and toxicity, investigators rely on treatment records alone to infer changes in clinical disease status and determine LOTs. This non‐uniform methodology may cause significant variation in how LOTs are defined depending on malignancy type, data source, and treatment patterns. Standardized rules and algorithms for determining LOTs are needed to improve the reproducibility and comparability of real‐world data analyses.

There is tremendous opportunity for impactful analysis of real‐world data from patients with metastatic non‐small cell lung cancer (mNSCLC), but standardized LOT determination has been difficult. Systemic therapies for mNSCLC now include immunotherapy, chemotherapy, and targeted therapies, which can be used alone or in combination. Because of this, treatment patterns and sequencing are complex and heterogeneous [1]. This is particularly true for the subset of patients with mNSCLC and rare molecular characteristics and patients with co‐morbidities where complex treatment patterns and outcomes are only captured outside of clinical trials.

Previously, others have proposed algorithms for determining LOTs for patients with solid tumors, lung cancer, and mNSCLC [2]. However, these algorithms do not specifically incorporate common treatment patterns for patients with targetable alterations, such as *EGFR*, *ALK*, and *ROS1* alterations. In this report, we aim to propose new rules for determining LOT from real‐world data of patients with mNSCLC, focusing on treatment patterns among patients receiving targeted therapies.

## 2 Methods

**FIGURE 1:**
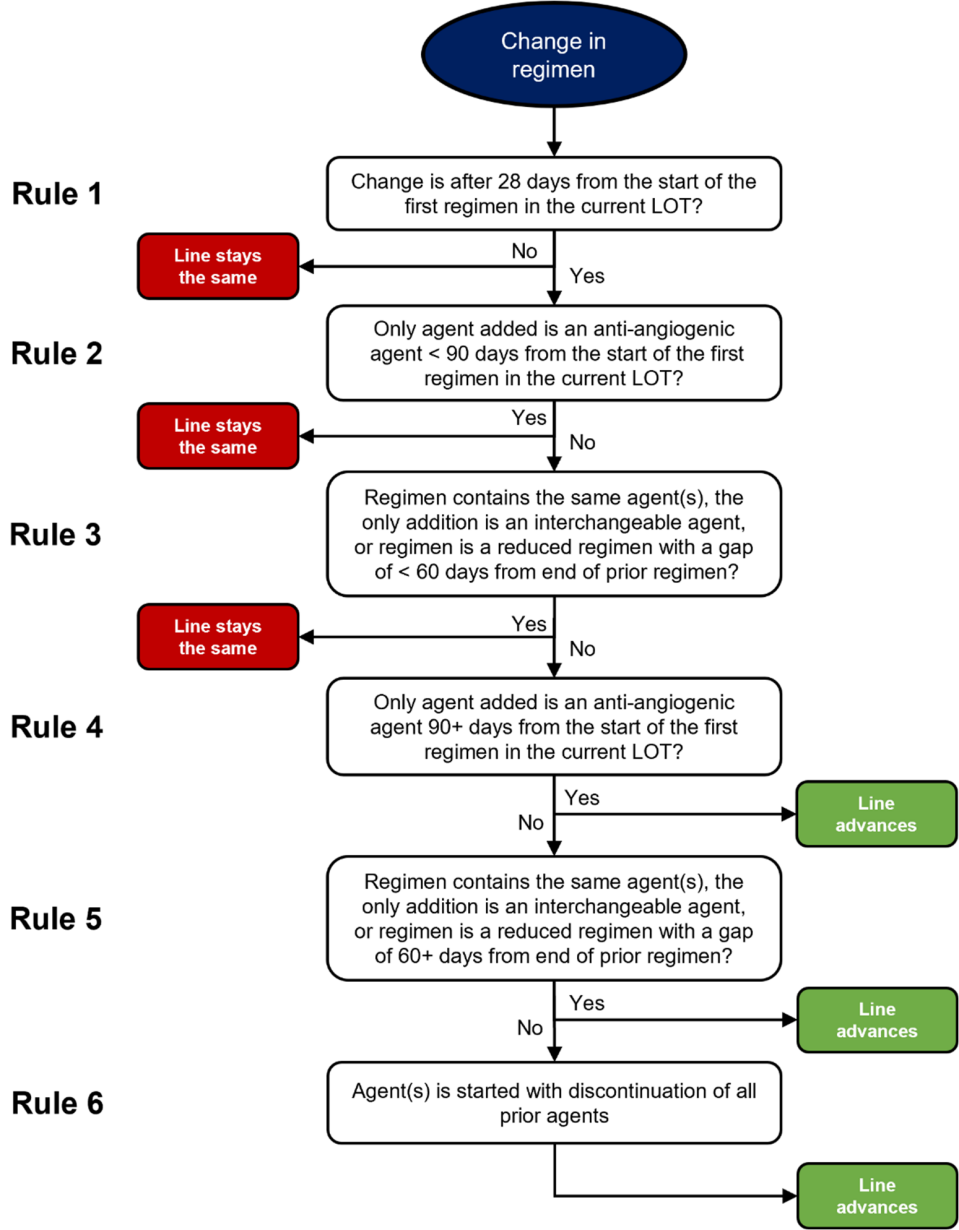
Algorithm for line of therapy determination.

We defined a regimen as any systemic anticancer agent (i.e., chemotherapy, immunotherapy, or targeted therapy) or combination of systemic agents administered at the same time. First, to develop rules for determining LOT (“LOT rules”), we reviewed existing algorithms designed to classify cancer treatments into LOTs and assessed their compatibility with contemporary treatment practices in mNSCLC (e.g., in the era of targeted therapies) [2, 3, 4]. Next, these rules were applied to treatment records from 550 patients with mNSCLC and sensitizing *ALK* or *ROS1* fusions in the Academic Thoracic Oncology Medical Investigators Consortium's (ATOMIC) Driver Mutation Registry. ATOMIC is a multi‐institutional collaborative of 12 academic centers across North America (11 United States, 1 Canada). The Driver Mutation Registry is an electronic medical record‐based, retrospective registry of patients with mNSCLC and *ALK*, *ROS1*, or *EGFR* alterations treated at ATOMIC sites [5, 6]. Trained data abstractors used a standardized digital form to record patients' regimen agent(s) and start/end dates. Clinical experts consisting of 23 thoracic oncologist members of ATOMIC (all agreed to be co‐authors) reviewed the LOT determination rules and their performance in the Driver Mutation Registry and proposed revisions to reflect practice patterns most indicative of changes to clinical disease status. Finally, these modified rules were reviewed and received unanimous approval. These rules for determining LOT in real‐world data of patients with mNSCLC are outlined in Table 1 and algorithmically indicated in Figure 1. To illustrate the performance of these rules, they were applied to a separate ATOMIC cohort of 1133 patients with mNSCLC and *EGFR* sensitizing mutations.

**TABLE 1:** Proposed rules for LOT determination from real‐world treatment regimens.

Rule	Line of therapy stays the same	Example regimen change	Example LOT determination	Number of times rule is applied in the ATOMIC *EGFR* cohort
1	If any agent is introduced within 28 days of the start of the first regimen of the current LOT	Day 0–21: Carboplatin, Pemetrexed Day 14–400: Brigatinib	0.5L^a^ (Day 0–21): Carboplatin + Pemetrexed 1 L (Day 14–400): Brigatinib	109
2	If an angiogenesis inhibitor (e.g., bevacizumab, ramucirumab) is introduced within 90 days from the start of the first regimen of the current LOT	Day 0–80: Osimertinib Day 80–200: Osimertinib, Bevacizumab	1 L (Day 0–200): Osimertinib + Bevacizumab	1
3	If the gap between the end of the prior and the start of the current regimens is ≤ 60 days and the current regimen uses the same agent(s), an exchange of interchangeable agents (e.g., Carboplatin/Cisplatin, Paclitaxel/Nab‐paclitaxel), or a reduced regimen	Day 0–30: Entrectinib Day 80–110: Entrectinib	1 L (Day 0–110): Entrectinib	224

Rule	Line of therapy advances	Example regimen change	Example LOT determination	
4	If an angiogenesis inhibitor (e.g., bevacizumab, ramucirumab) is introduced > 90 days after the start of the first regimen of the current LOT.	Day 1–120: Osimertinib Day 121–160: Osimertinib + ramucirumab	1 L (Day 1–120): Osimertinib 2 L (Day 121–160) Osimertinib + ramucirumab	22
5	If the gap between the end of the prior and the start of the current regimens is > 60 days and the current regimen uses the same agent(s), an exchange of interchangeable agents (e.g., Carboplatin/Cisplatin, Paclitaxel/Nab‐paclitaxel), a reduced regimen	Day 1–200: Alectinib Day 300–400: Alectinib	1 L (Day 1–200): Alectinib 2 L (Day 300–400): Alectinib	34
6	If a new non‐interchangeable agent is added to a regimen or is introduced after the discontinuation of all prior agents > 28 days after the start of the first regimen of the current LOT.	Day 1–42: Carboplatin + pemetrexed Day 43–700: Lorlatinib	1 L (Day 1–42): Carboplatin + pemetrexed 2 L (Day 43–700) Lorlatinib	1645

Abbreviation: LOT, line of therapy.

^a^ When a regimen is discontinued followed by the start of a new regimen all within 28 days of the start of the first line of therapy, the first regimen is assigned a line number of 0.5 while still being considered part of first‐line therapy.

Additional rules were developed under the same process to identify LOTs that are most likely applicable to early‐stage disease (“ESD rules”) since data sources of patients with mNSCLC may inadvertently include treatments for this disease stage. We developed two rules to remove systemic therapy agents likely used in the treatment of early‐stage disease from the LOT determination. (1) If a LOT was durvalumab monotherapy administered prior to November 11, 2022 (i.e., prior to Food and Drug Administration approval for durvalumab in stage IV NSCLC) [7] or (2) if the LOT was chemotherapy with a cumulative cycle duration of 30–140 days and no subsequent LOT for at least 4 months of follow‐up, the LOT should be considered as a treatment for early‐stage disease and excluded from the patient's LOT enumeration for mNSCLC.

In this report, we demonstrate the use of LOT and ESD rules on treatment records from the ATOMIC *EGFR* cohort. We compared our LOT determination with those of an existing algorithm for mNSCLC proposed by Hess et al. using the SAS macro %mnsclc_lot [2]. Analyses were performed in R version 4.2.1 [8] and SAS software version 9.4.

## 3 Results

Among 1133 patients in ATOMIC with *EGFR* alterations and mNSCLC, a total of 3168 regimens were recorded with a median of 2 regimens per patient (IQR, 1–4; range, 1–13). After applying our six LOT rules, there were 2834 total LOTs with a median of 2 LOTs per patient (IQR, 1–3; range, 1–11). Rules 1–3 prevented 334 regimen changes from advancing the LOT (Table 1). Thirty‐seven patients switched regimens within 28 days of the start of the first LOT and had a line 0.5 recorded.

Comparatively, after applying an algorithm proposed by Hess et al. to the same treatment records, there were 2771 total LOTs with a median of 2 per patient (IQR 1–3; range 1–9). Among our method's 3999 agent‐LOT assignments (e.g., osimertinib as 1 L and carboplatin as 2 L), ours differ from the Hess method in 5.7% of assignments. Examples of patients with these differences are shown in Table 2.

**TABLE 2:** Example LOT determination differences between the proposed rules and other (Hess) rules.

Patient	Duration of regimen (days)	Regimen agent(s)	Proposed LOT	Hess LOT
Patient A	0–150	Erlotinib	1	1
	151–242	Osimertinib	2	2
	243–438	Bevacizumab, Carboplatin, Pemetrexed	3	3
	439–674	Pemetrexed	3	3
	675–702	Bevacizumab, Pemetrexed	4	3
	703–849	Pemetrexed	4	3
	881‐ongoing	Bevacizumab, Erlotinib	5	3
Patient B	0–630	Bevacizumab, Erlotinib	1	1
	632–1036	Osimertinib	2	2
	1038–1091	Osimertinib, Crizotinib	3	2
Patient C	0	Bevacizumab, Carboplatin, Pemetrexed	1	1
	30–800	Erlotinib	2	2
	802–904	Bevacizumab, Carboplatin, Pemetrexed	3	3
	905–1010	Bevacizumab, Pemetrexed	3	3
	1205–1849	Osimertinib	4	4
	1851–2035	Osimertinib, Gefitinib	5	4

ESD rules aimed to identify treatments for early‐stage disease. Among patients in ATOMIC with *EGFR* alterations and mNSCLC, the first LOT for 81 patients met the criteria for early‐stage disease treatment (ESD Rule 1 met for two patients and ESD Rule 2 met for 79). These LOTs were removed, and the remaining LOTs were renumbered as LOTs for mNSCLC.

## 4 Discussion

We propose six simple rules to determine whether a change in treatment for mNSCLC should or should not advance the LOT in real‐world data. Our LOT assignments led to different classifications from previous work in 5.7% of cases, primarily in characterizing targeted therapy‐containing regimens. This difference is expected to be larger in datasets where targeted therapies are more common.

Valid LOT determination from treatment records is consequential because it can affect outcome measures. For instance, consider a time to treatment discontinuation (TTD) outcome that defines starting a new LOT as the event of interest and censors at last treatment otherwise. For the TTD of the second LOT, Patient B (Table 2) under our rules would have a TTD of 407 days, but under prior rules, they would be censored at 460 days. More generally, because our LOT rules more frequently advance the LOT compared to a prior method, we would expect shorter time‐to‐events using our rules (i.e., lower failure‐free probability), all else being equal. Therefore, it is critical that the LOT determination accurately reflects clinical practice. Future research may consider the sensitivity of outcomes to various algorithms of LOT determination and consider modifications to LOT rules as treatment patterns and paradigms evolve.

There are several ways that our proposed rules better characterize LOTs in mNSCLC populations treated with targeted therapy compared to previously reported methods (Table S1) [2, 4, 5, 6, 7]. First, we distinguish between biologics and therapies targeting oncogenic drivers in mNSCLC. This distinction is important because biologics, such as angiogenesis inhibitors, may have little anti‐tumor activity alone and are often added or removed from treatment regimens without a clear change in disease status, while targeted therapies have tremendous anti‐tumor activity and are used in the setting of a change in disease status. Second, there are instances where targeted therapies are added to or replace a previous regimen because of a newly identified targetable mutation and not because of a change in disease status. The rules proposed allow for this approach by allowing for 1 cycle of chemotherapy to be given while waiting for molecular testing results prior to switching to a targeted therapy without advancing the LOT. Third, we allow for a treatment pause and re‐initiation within 60 days to be considered the same LOT, which may be more appropriate for targeted therapies typically taken daily by mouth that are paused and restarted for toxicity [3, 9, 10].

Retrospective determination of whether a treatment was given in the setting of metastatic disease versus early‐stage disease is critical to determining real‐world clinical endpoints. In this historical dataset, we used rules based on the approved therapies at the time of the dataset, but even while completing the analysis for this study the indications for several systemic therapies expanded to include early‐stage disease, making the agent itself an unreliable indicator of early‐stage treatment. Given this complexity, extracting the date of metastatic diagnosis at the time of data abstraction will become even more important to evaluate the practice patterns contemporary to the time of the dataset.

We propose a simple algorithm to classify treatment regimens into LOTs. Use of these rules provides a transparent approach to LOT assignment, in keeping with the goals of research transparency of the International Society of Pharmacoepidemiology's Guidelines for Good Pharmacoepidemiology Practice [11]. We believe wider use of this framework for LOT determination could lead to better method harmonization and transparency of observational studies in mNSCLC and allow for comparison and aggregation across datasets in the future.

## Conflicts of Interest

C.B.G., W.‐T.H., A.C., G.L., D.P., G.L.B.M., A.H., W.S., J.D., K.L.M., V.V., D.R.C. declare no conflicts of interest. J.E.R.: Advisory Board/Consultant: Genentech/Roche, Sanofi/Genzyme, Personalis, Guardant, AstraZeneca, BMS, Arcus, AbbVie, Daiichi Sankyo. Research Funding (to institution): Genentech/Roche, Verastem, Nuvalent, Mesothelioma Applied Research Foundation, LUNGevity Foundation. Honoraria/Speaking Fees: AstraZeneca, Merck. W.I.: Consultant for OncLive, Clinical Care Options, Chardan, Cello Health, Curio Science. Advisory Board/DSMC for Genentech, Mirati, Outcomes Insights, Jazz Pharmaceuticals, GI Therapeutics, Takeda, AstraZeneca, Sanofi, Janssen, Amgen, Bristol Myers Squibb, Novocure. S.V.L.: Advisory Board/Consultant for AbbVie, Amgen, AstraZeneca, Boehringer Ingelheim, Bristol‐Myers Squibb, Catalyst, Daiichi Sankyo, Eisai, Elevation Oncology, Genentech/Roche, Gilead, Guardant Health, Janssen, Jazz Pharmaceuticals, Merck, Merus, Mirati, Novartis, Regeneron, Sanofi, Takeda, Turning Point Therapeutics. Research grant (to institution) from Alkermes, Elevation Oncology, Genentech, Gilead, Merck, Merus, Nuvalent, RAPT, Turning Point Therapeutics. Data Safety Monitoring Board for Candel Therapeutics. T.P.: Advisory Role (advisory boards or consultations) in last 3 years: AstraZeneca, Biocept, Boehringer Ingelheim, Bristol‐Myers Squibb, Bicara, Caris, Guardant Health, Guidepoint, EMD Serono, Janssen, Jazz Pharmaceuticals, Mirati Therapeutics, Natera, Pfizer, Sanofi, Regeneron, Roche/Genentech, Takeda. Advisory Committees: Elevation Oncology (DSMB). Research Funding: EMD Serono, Janssen, Gilead. J.J.N.: Stock/Ownership interests in Epic Sciences, Cansera, Quantagene, Indee P/L. Consulting for AstraZeneca, Naveris, Aadi biosciences, Atla, MindMed, ANP Technologies. Research funding to institution from Merck, Genentech. Patent pending on movement and unexpected healthcare encounters. K.A.M. has received consulting/advisory fees from AstraZeneca, Amgen, Janssen, Mirati Therapeutics, Daiichi Sankyo/Lilly and Puma Biotechnology, as well as Honoraria from AstraZeneca. KAM receives research funding to Johns Hopkins University from Bristol‐Myers Squibb and Mirati Therapeutics. V.K.L. reports serving in a consultant/advisory role for Seattle Genetics, Bristol‐Myers Squibb, AstraZeneca, Guardant Health, Takeda, and AnHeart Therapeutics, and has received research funding from BMS, Merck, Seattle Genetics, Astra Zeneca. L.C.V. reports researching funding from Janssen, BMS, Merck, Regeneron, GSK, AstraZeneca, BioAtla, Black Daimon Therapeutics, Jazz, Genentech, BeiGene. Consulting fees from Takeda, Janssen, InterVenn Biosciences, Sanofi, Daiichi Sankyo, Jazz, BMS, Gilead. C.A. reports receiving institutional research funding from AstraZeneca, Genentech, Incyte, MacroGenics, Medimmune, and Merck Sharp & Dohme, and receiving consultation fees from Genentech, Lilly, Celgene Merck, AstraZeneca, Blueprint Genetics, Shionogi, Daiichi Sankyo/Astra Zeneca, Regeneron/Sanofi, Eisai, BeiGene, Turning Point, Pfizer, Janssen, Boehringer Ingelheim. L.S. reports research funding from Blueprint (Inst), Seagen (Inst), IO Biotech (Inst), Erasca (Inst). Consulting with Sanofi Genzyme, Regeneron, Genmab, Seagen, and Bayer. M.E.M. reports researching funding from Eli Lilly (Inst), AstraZeneca (Inst), Merck (Inst), Genentech (Inst) consulting role with Astra Zeneca, Novocure, Boehringer Ingelheim, Janssen, Takeda, Blueprint Pharmaceuticals, Bayer, Bristol Myers Squibb, Ikena; Honorarium from Thermo Fisher; stock in Merck, Johnson & Johnson.

## Supporting information


**Table S1.** Differences between proposed rules and Hess et al. rules.
