# Supplementary material for: Determining Line of Therapy from Real‐World Data in Non‐Small Cell Lung Cancer
Source: Pharmacoepidemiol Drug Saf. 2024 Nov 25;33(12):e70049. doi: 10.1002/pds.70049 (PMC11588435; doi:10.1002/pds.70049)
Supplement: Supplementary file 1 — Table S1. Differences between proposed rules and Hess et al. rules. [file PDS-33-e70049-s001.docx]

**Supplemental Table 1**: Differences between proposed rules and Hess et al. rules

| Scenario | LOT advances | |
| --- | --- | --- |
|  | Proposed rules | Previous (Hess) rules |
| Targeted or immunotherapy is added to a regimen 28+ days after the start of the first regimen in the current LOT. | Yes | No |
| Targeted or immunotherapy is started with discontinuation of all agents in the prior regimen. | Yes*  *Except if started <28 days from start of LOT | Yes |
| An interchangeable agent is exchanged 60+ days after the end of the prior regimen. | Yes | No |
| Introduction of an anti-angiogenic agent <90 days from the start of any LOT, with or without discontinuation of the prior regimen. | No | Yes*  *Except if pemetrexed is part of prior regimen and given with anti-angiogenic agent in the first LOT |
| A regimen is reduced but with a gap in treatment of 60+ days.  (e.g., Day 0-60: Carboplatin + Alectinib  Day 140-ongoing: Alectinib) | Yes | Not addressed in rules |
